# Improving Blueberry Fruit Nutritional Quality through Physiological and Genetic Interventions: A Review of Current Research and Future Directions

**DOI:** 10.3390/antiox12040810

**Published:** 2023-03-26

**Authors:** Priti Krishna, Gareema Pandey, Richard Thomas, Sophie Parks

**Affiliations:** 1 School of Science, Western Sydney University, Locked Bag 1797, Penrith, NSW 2751, Australia; 2 Graphic Era University, Clement Town, Dehradun 248002, Uttarakhand, India; 3 Department of Primary Industries, Central Coast Primary Industries Centre, Locked Bag 26, Gosford, NSW 2250, Australia

**Keywords:** antioxidants, blueberry, flavonoid, anthocyanins, polytunnels, hydroponics

## Abstract

Blueberry, hailed as an antioxidant superfood, is the fruit of small shrubs in the genus *Vaccinium* (family Ericaceae). The fruits are a rich source of vitamins, minerals and antioxidants such as flavonoids and phenolic acids. The antioxidative and anti-inflammatory activities derived from the polyphenolic compounds, particularly from the abundantly present anthocyanin pigment, have been highlighted as the major contributing factor to the health-benefitting properties of blueberry. In recent years, blueberry cultivation under polytunnels has expanded, with plastic covers designed to offer protection of crop and fruit yield from suboptimal environmental conditions and birds. An important consideration is that the covers reduce photosynthetically active radiation (PAR) and filter out ultraviolet (UV) radiation that is critical for the fruit’s bioactive composition. Blueberry fruits grown under covers have been reported to have reduced antioxidant capacity as compared to fruits from open fields. In addition to light, abiotic stresses such as salinity, water deficit, and low temperature trigger accumulation of antioxidants. We highlight in this review how interventions such as light-emitting diodes (LEDs), photo-selective films, and exposure of plants to mild stresses, alongside developing new varieties with desired traits, could be used to optimise the nutritional quality, particularly the content of polyphenols, of blueberry grown under covers.

## 1. Introduction

An increasing awareness of the benefits of a healthy lifestyle is providing impetus for healthy eating, which among other options includes a higher intake of fruits and vegetables. The incorporation of nutrient-rich fruits in the diet can provide immunity against certain diseases and reduce the risk of acute or chronic ailments [1,2]. Cognizance of the health benefits of such fruits has elevated consumer demand [3], resulting in an overall market growth for these fruits [4], valued at USD 152 billion in 2021 globally, with a further 41% increase expected by 2027 [5]. Blueberry is one of the most nutrient-dense berries, packed with fibre, vitamins and antioxidant components that belong to the wide group of flavonoids and phenolic acids with functional properties that decrease the risk of heart disease, diabetes and neurodegeneration [6,7,8]. Consumer demand for blueberry has soared globally, leading to increased production at an average annual rate of 6.1% [9]. In 2021, the global blueberry industry was worth USD 938 million [10], with China as the leading highbush blueberry producer (477,080 tonnes), followed by the United States (328,210 tonnes). The blueberry planting area also increased by 12.1% and is expected to increase by 25.3% in upcoming years [11].

Blueberries are deciduous shrubs of the genus *Vaccinium* (family Ericaceae; subfamily Vacciniaceae). The genus *Vaccinium* comprises about 450 species, including cranberry (*V. macrocarpon* Ait.), bilberry (*V. myrtillus* L.), lingonberry (*V. vitis-idaea* L.), and huckleberry (*V. parvifoium*), which are valued for their sweet flavoured and nutrient-rich fruits [12]. Considering the health benefits of blueberries and their escalated demand worldwide, it is widely accepted that the production and sales of blueberries will continue to increase steadily for the foreseeable future. Fruit quality traits draw premium prices for producers while also influencing consumer preferences. Important blueberry traits for consumers, and therefore for breeders, include freshness, flavour, texture (firmness), and shelf life [13,14]. Another key focus is to improve fruit nutritional quality by increasing bioactive compounds content [15], which is strongly influenced by environmental factors such as light, temperature and water availability [16,17]. Knowledge of the environmental factors and growing conditions that positively influence bioactive accumulation will provide an opportunity to develop agronomic practices that can enhance the nutritional quality of blueberry fruits. This review draws upon basic and applied knowledge to suggest agronomic, physiological, and genetic interventions for the current growing practices of blueberries that can enhance the health-benefitting nutritional qualities of the fruit, in addition to traits such as flavour, texture, and size.

## 2. Blueberry Species and Characteristics

The most common blueberry species are the wild-growing lowbush blueberry (*V. angustifolium*), the cultivated highbush blueberry (*V. corymbosum*), and the rabbiteye blueberry (*V. virgatum*, syn. *V. ashei*) [18]. The various species differ in their soil and climatic requirements. For example, lowbush blueberry is tolerant of a wide range of temperatures and is common in abandoned pastures. In contrast, northern highbush blueberries are limited to cold temperature regions, while the southern highbush, which are interspecific hybrids of *V. corymbosum*, and *V. angustifolium*, *V. darrowii*, *V. ashei*, *V. tenellum* and others, are suited to warmer regions [19]. The rabbiteye blueberry, native to the southeast US, is more drought and heat tolerant than both lowbush and highbush blueberries [18]. The commercialisation of blueberries has relied on developing hybrids between the tetraploid lowbush *V. angustifolium* (2n = 4x = 48), tetraploid highbush *V. corymbosum* L. (2n = 4x = 48), and the hexaploid rabbiteye *V. virgatum* (2n = 6x = 72) [20]. Although most blueberry breeding efforts have focussed on soil and climate adaptation, early bloom, and fruit traits such as size, firmness, and sweetness, due to the high labour cost of harvesting, the blueberry industry’s current trait priorities are bush architecture, loose fruit clusters and easy detachment of mature fruit that will allow for machine harvesting [21,22].

The blueberry plant has underground stems known as rhizomes and shallow roots of two types: thick storage roots that anchor the plant, and thin thread-like roots that perform the function of water and nutrient absorption. Shoots emerge from buds located in the crown region. In a one-year-old blueberry plant, the shoot has inflorescence buds at the top and vegetative buds at the lower part. The simple leaves appear in an alternate arrangement on the branches. The inflorescence bud can produce 9–10 flowers at the tip, with numbers reducing as the distance from the tip increases. Following floral growth and pollination, the fruit develops as a true berry with many seeds [18]. The raw green fruit turns reddish-purple during the ripening phase and eventually dark purple during the harvesting time. The skin of the fruit is covered with a waxy bloom which protects the fruit from moisture loss and decay, maintaining the postharvest quality [23]. The fruit has a sweet and tangy flavour that fluctuates based on the sugar/acid ratio [24]. The various aspects of blueberry development can vary greatly in different cultivars and in response to environmental conditions.

## 3. Major Phytochemicals in Blueberry Fruits and Leaves

Often referred to as a superfruit, blueberries are rich in dietary fibre (2.4–3.5% of fruit weight), vitamins (C and K, and low levels of A, B and E) [7,25,26], minerals (calcium, iron, magnesium, manganese and zinc) [27,28] and bioactive compounds containing polyphenols (anthocyanins and flavonols) [29], phenolic acids [30] and carotenoids (lutein) [26,29]. Plant phenolics and polyphenols are secondary metabolites that act as defence compounds against environmental stresses such as high light, UV, unfavourable temperatures, water deficit, salinity, nutrient deficiencies, pathogen infection and herbivores [31]. These environmental stresses can lead to increased production of free radicals and other oxidative species that attack important macromolecules such as DNA, proteins and lipids, resulting in cell damage and homeostatic disruption. Many plant phenolic compounds have been found to scavenge free radicals and therefore act as antioxidants [6,7,8,29,30,32]. Acknowledged as natural antioxidants, plant phenolics have gained popularity for their pharmacological properties such as anti-inflammatory, anticancer, antimicrobial, antiallergic, antiviral, antithrombotic, hepatoprotective and others [33,34]. The polyphenols of the flavonoid group are subdivided into various subgroups such as neoflavonoids, flavones, flavonols, flavanones, flavanonols, flavanols, catechins, anthocyanins, and chalcones [35].

The polyphenol content in blueberries ranges from 48 to 304 mg/100 g of fresh fruit weight, with the anthocyanin flavonoids (malvidin, delphinidin, petunidin, cyanidin, and peonidin) accounting for up to 60% of the total polyphenolics in ripe blueberries [36]. In addition to anthocyanins, flavonoid subgroups flavonols (kaempferol, quercetin, myricetin) and flavanols (proanthocyanidins) as well as phenolic acids (mainly hydroxycinnamic acids) contribute to health-benefitting properties of blueberry [37,38]. Table 1 summarizes the bioactive constituents of blueberry fruit. According to studies of blueberry fruit chemical composition, the polyphenol content varies as a function of genetics, environmental conditions and fruit ripening stages [36]. A study of 13 commercially important blueberry cultivars indicated that the fruits of these cultivars varied three-fold in total anthocyanin content, ranging from 94.5 to 301.0 mg/100 g fresh weight [39]. Similarly, 90% of the genotypes among a selection of 80 highbush and 135 lowbush genotypes spanned a 1.6-fold range in fruit anthocyanin content [40].

**Figure 1:**
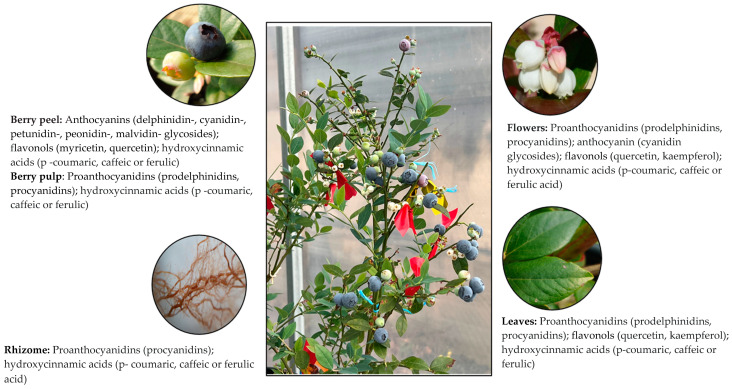
Bioactive compounds present in blueberry plant parts (text based mainly on values reported by [42]).

In addition to the fruit, other parts of the blueberry plant also accumulate bioactive compounds (Figure 1). The phenolics composition in blueberry leaves varies during the seasons and is reflected in the change of leaf colour from green to red in autumn [41]. Although flavonols (quercetin, kaempferol), hydroxycinnamic acids (p-coumaric and caffeic or ferulic acids) and proanthocyanidins (prodelphinidins and procyanidins) were present in both red and green leaves, with the exception of proanthocyanidins, all other components were present in significantly higher amounts in red leaves [42,43,44]. Chemical profiling of leaves from six commercial blueberry (*V. corymbosum* L.) varieties indicated that flavanols and flavonols were the most abundant phenolic subclasses, with rutin as the major compound present [44]. The seasonal increase in phenolic acid and anthocyanin content in blueberry leaves was correlated with an increase in the antioxidant activity of blueberry leaves [45].

The biological and therapeutic uses of blueberry leaves (reviewed in [44]) range from preventing body fat accumulation in mice [46], improving mitochondrial function [47], and providing neuroprotective effects [48] to possessing antimicrobial antimutagenic activity [44]. Some studies indicate that blueberry leaves contain higher levels of phenolic compounds and exhibit higher bioactivity as compared to the fruit [49,50]. With blueberry cultivation on the increase, the leaf biomass could be used in functional foods and as a dietary supplement.

The main phenolic compounds in blueberry rhizomes were identified as procyanidins and hydroxycinnamic acids, and in flowers as quercetin and hydroxycinnamic acid [42]. Compared to other parts, blueberry roots have very low amounts of procyanidin, catechin and epicatechin, chlorogenic acid, and caffeic acid [51]. From studies to date, blueberry fruits and leaves have emerged as the most promising sources of bioactive compounds with antioxidant properties.

**Table 1:** Bioactive constituents of blueberry fruit.

Nutritional Composition			(mg/100 g)	References
Vitamins	Vitamin C		3.4–9.5	[25]
	Vitamin B complex	Vitamin B1	19.6–26.7	[25]
		Vitamin B2	38.0–70.2	[25]
		Vitamin B3	1.0–1.7	[25]
		Vitamin B6	0.052	[52]
	Vitamin E		0.57	[52]
	Vitamin K		56.1–79.9	[25]
	Vitamin A		5.0–83.1	[25]
Macro-elements	Nitrogen		74.4–103.1	[28]
	Calcium		6.6–15.2	[28]
	Magnesium		4.5–10.1	[28]
	Potassium		66.2–98.0	[28]
	Phosphorus		6.8–20.3	[28]
	Sulphur		10.1–25.4	[28]
Micro-elements	Iron		0.15–0.57	[25]
	Manganese		0.14–1.52	[28]
	Copper		0.01–0.09	[28]
	Boron		0.08–0.14	[25]
	Molybdenum		0.003–0.012	[28]
	Zinc		0.06–0.13	[25]
Total Phenolic Content			393 ± 52	[52]
Total Flavonoids			2.5–387.48	[53]
	Anthocyanidins (mg/kg FW)		134	[54]
	Anthocyanins		233 ± 34	[52]
		Malvidins	22–33%	[53]
		Delphinidins	27–40%	[53]
		Petunidins	19–26%	[55]
		Cyanidins	5.7–14%	[55]
		Peonidins	1.4–4.5%	[55]
	Flavonols (mg/kg FW)		38–46	[52,54]
	Quercetin		24	[56]
	Myricetin		26	[56]
	Flavanols (mg/kg FW)		1.1	[54]
Carotenoids		Lutein	1.53	[26]

## 4. Health Benefits of Blueberry Consumption

**Figure 2:**
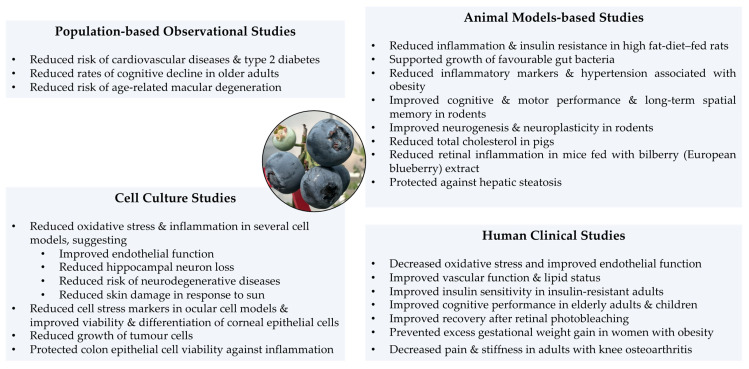
Health benefits of blueberry consumption. The most significant health-related benefits of blueberry are based on the antioxidant and anti-inflammatory actions of the phytochemicals, mainly anthocyanin, contained in the fruit [6,7,8,30,59,60,61,62,63].

The health benefits of blueberry consumption, either as fruit or products derived from the fruit, have been evidenced from human population observational and clinical research, as well as research using animal models and cell lines as biological models (Figure 2). While the synergistic effect of the numerous phytochemicals present in blueberries is likely responsible for the health benefits, the antioxidative and anti-inflammatory activities derived from the polyphenolic compounds, particularly from the abundantly present anthocyanin pigment, have been highlighted as the contributing factors in most studies. A wild blueberry drink intake for six weeks, as compared to the placebo drink, significantly reduced the levels of endogenously oxidized DNA bases and of H_2_O_2_-induced DNA damage, indicating the antioxidative potential of blueberry [57]. A review of eight clinical trials and 23 animal studies indicated that blueberry consumption significantly reduced oxidative stress in both humans and rodents, concomitantly improved endothelial function in humans, and improved glucose tolerance while reducing triglyceride levels in rodents [58].

Numerous research articles and reviews have been published on the health benefits of blueberries over the last decade. The most significant correlations that have emerged from research involving human and animal studies are between high blueberry intake and reduced risk of heart disease, type 2 diabetes and visual deterioration, less weight gain and slower rates of cognitive decline (reviewed in [6,7,8,30,59] and references therein). A systematic review and meta-analysis investigating the effect of blueberry intervention on metabolic syndrome risk factors revealed that blueberry intervention decreased total cholesterol and low-density lipoprotein (LDL) levels as well as diastolic blood pressure [60]. An investigation into blueberry effects in adults suffering from knee osteoarthritis revealed that daily incorporation of blueberries in diet reduced pain and stiffness and improved functionality [61]. Using colon epithelial cell lines stimulated with a proinflammatory cytokine cocktail as an in vitro inflammatory bowel disease model, Driscoll et al. [62] demonstrated significant decreases in reactive oxygen species (ROS) and increases in cell viability following treatment with blueberry extract. The effects of blueberry on cell models of immune, endothelial and vascular, brain and neuronal, dermal, ocular and intestinal systems have been recently reviewed [63]. These studies indicate that blueberry reduces oxidative stress and inflammation by downregulating the NF-κB pathway and by reducing the levels of ROS and lipid peroxidation. In summary, the interest in blueberry as a superfood is clearly founded on a body of scientific evidence which is continuing to grow. With the broad spectrum of therapeutic potential (Figure 2), there is little doubt that this super fruit will drive food innovation for the development of blueberry-based functional foods and nutraceuticals in the future.

## 5. Blueberry Production Systems

**Figure 3:**
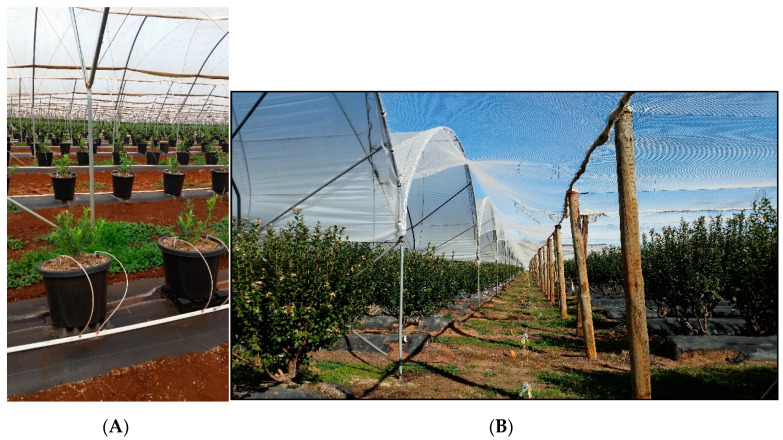
(**A**). Blueberry crop grown in a substrate under a polytunnel with water and nutrients supplied through fertigation. (**B**). A soil-grown blueberry crop protected by plastic cover (**left**) and bird net (**right**).

In recent years, polytunnels covered with plastic covers (Figure 3A) or bird and shade nets (Figure 3B) have become popular for growing soft fruits and certain vegetables to prevent crops from excessive radiation, wind, hail frost and birds [64]. Blueberry has specific requirements in terms of soil texture, temperature, pH and quality of water used for irrigation. To obtain homogeneity and protection from environmental conditions and pests, blueberry cultivation has increasingly moved from growing in soil in the open to hydroponics cultivation, also known as soilless cultivation, under plastic covers (Figure 3A). Growing blueberries in hydroponic systems allows for better control over water and nutrients, better root growth, and an increase in plantation density. There are several other benefits of growing crops under covers. For instance, blueberry production is increased under covers due to early bud development [65], higher temperatures under tunnels enhance fruit setting [66], and diffusion of light favours leaf stomatal conductance and enhances photosynthesis [65,66,67]. In addition, higher productivity for the retail market is achieved by extending the growing seasons under crop covers [68].

The disadvantage for cultivation under covers is that some covers can reduce 30–40% transmission of photosynthetically active radiation (PAR) [66,69]. Reduction in PAR and changes in the spectral quality of light received by plants can reduce the photosynthetic rate and trigger shade-avoidance responses, causing shoot elongation [70], all of which adversely affect yield and fruit quality [69,71]. Furthermore, crop covers that contain UV stabilizers or absorbers to reduce polyethylene degradation block plant exposure to UV. Although the blueberry plant appears to maintain the photosynthetic rate even with a 40% reduction in PAR [72], an important consideration is how the covers that filter out UV radiation (260–400 nm), a critical wavelength for increasing fruit bioactive composition, affect the nutritional quality of the blueberry fruit. Blueberry fruits grown under covers had reduced total flavonols and antioxidant capacity as compared to fruits from open fields [73,74], and blueberry plants grown under coloured photo-selective nets (blue, white, and red) produced larger and heavier berries but with lower anthocyanin levels compared to berries grown in full sunlight [75]. The black photo-selective net, which produced shading at 90%, negatively impacted growth, ripening and anthocyanin accumulation [75].

## 6. Light as a Regulator of Phytochemical Content with Antioxidant Activity

Light is a key factor that affects growth, colour, ripening and quality, including the nutritional value, of fruits. Plants respond to light quantity (flux), quality (wavelength), duration (photoperiod) and direction (tropism) during their developmental cycle, from germination, through transition, to flowering and fruit ripening. This light-mediated development in plants is termed photomorphogenesis [76]. Plants perceive light through different families of photoreceptors: phytochromes (red/far-red light receptors, 600–750 nm), cryptochromes and phototropins (blue/UV-A light receptors, 320–500 nm) and UV-B photoreceptors (UVR8, 282–320 nm). These photoreceptors act either alone or in combination with each other to regulate the various aspects of plant growth and development [77,78]. For photosynthesis, plants utilize only a portion of the solar light spectrum that lies in the range of 400 to 700 nm wavelength known as PAR [79]. However, variable ratios of wavelengths, including UV (250–400 nm) and far-red (700–800 nm), can control certain developmental and physiological responses in plants [80,81], such as altering the seed germination phase, flower development [82] and fruit quality [83]. For example, a red:far-red ratio of less than 1 increased chlorophyll content and the number of inflorescences in roses [84] and enhanced anthocyanin levels in cranberry fruits [85]. A red:blue ratio of 3 produced a high yield and quality in sweet basil grown indoors [86], and a combination of red and blue lighting enhanced flavonoid content in seedlings of the medicinal plant *Anoectochillus roxburghii* [87].

The effective spectral wavelengths of UV can induce a stress response in plants by triggering the formation of free radicals (ROS) that cause oxidative damage. To protect the photosynthetic apparatus from damage, plants synthesize UV-absorbing compounds such as flavonoids and carotenoids. In addition to having free radical scavenging activity, these compounds can absorb radiation in the UV range [88]. For this reason, UV, mainly UV-B radiation, has been used as an elicitor of flavonoid accumulation [88,89,90] and antioxidant properties in various crops [91,92,93,94], as well as of other abiotic and biotic responses in plants that can provide cross-tolerance against light, temperature and drought stresses [95,96]. UV-B also interacts with PAR to influence developmental processes such as seedling growth, leaf development and fruit ripening [78], as well as flavonoid accumulation [97].

The effects of UV and other wavelengths of light on polyphenolic production are highly dependent on plant species. In leafy greens, UV-A and UV-B induce the synthesis of anthocyanins, whereas the effect of blue light is varietal dependent [98], while in strawberries, a combination of UV-A, UV-C and blue wavelengths was seen to stimulate flavonoid biosynthesis [99,100]. Since sunlight is the most important environmental factor for flavonoid biosynthesis and accumulation, plants grown under covers, especially those that block UV, are likely to be affected in terms of flavonoid accumulation. Exceptions are some fruit species, such as tropical mangosteen, where flavonoid biosynthesis is primarily under developmental control [101].

In addition to light wavelength, an optimal intensity (flux) of light is required to maximize photosynthesis. Whilst high light intensity can damage the photosynthetic apparatus, low light levels starve the plant of chemical energy, leading to reduced productivity [102].

## 7. Light Interventions to Enhance Antioxidant Capacity in Fruits

Since PAR, UV, and their interaction can impact plant growth and fruit traits, light interventions have been used to enhance the antioxidative potential of fruits. A combination of red and UV light (280–330 nm) in polytunnels, for instance, stimulated anthocyanin accumulation in apple fruits [103] and increased the total flavonoid and phenolic content in tomatoes [104]. Flavonol accumulation in the leaves of the model plant Arabidopsis in response to a 6 h UV-B exposure interrupted with 0.5 h of recovery was higher as compared to continuous exposure of 6 h, indicating that discontinuous stress treatments could be developed for greenhouse cultivation practices [105].

Blueberry also responds to pre- and post-harvest exposure to UV by increasing flavonoid accumulation and the antioxidant capacity of fruits [106]. UV enhances flavonol accumulation during early fruit development and anthocyanin and proanthocyanidin contents during late fruit development. Blueberry grown in a greenhouse responded to UV-B with faster and uniform fruit maturation, higher accumulation of sugars and up to 167% increase in mature fruit anthocyanins [107]. Cultivar-dependent responses in blueberry leaves were noted, whereby UV-B-resistant cultivars were seen to accumulate faster and reach higher levels of antioxidants as compared to sensitive cultivars [108,109]. Exposure of blueberries to UV-C has predominantly been used for fruit sterilization [110], although the application extends to increasing the antioxidant properties of the fruit [93].

To obtain the maximum desired morphological or physiological effects in crops, artificial light sources such as light-emitting diodes (LEDs) of specific wavelengths and intensity are increasingly being used in various combinations in indoor farming [111]. There is very little information on the impact of LED lights on blueberry fruit quality. A few studies on the propagation of blueberries have indicated positive effects of LED-grown lighting on ex vitro development of highbush blueberry plants [112] and of mixed LEDs, particularly 50% red plus 50% blue light, on the growth of blueberry shoots and roots under aseptic and non-aseptic conditions [113]. A blue:red ratio of 1:2 enhanced the blueberry seedling growth parameters that are desirable for large-scale cultivation [114], whereas violet LED reduced blueberry plant growth but increased polyphenol and proline content in leaves [115].

Another emerging practice is the use of photo-selective covers, which along with the benefits of conventional covering, combine spectral manipulation to achieve desired crop responses. The covers are designed to selectively screen out non-optimal sunlight while transforming direct light into optimal and diffused light [116]. This improves penetration of the spectrally modified light into the inner plant canopy, thereby increasing the efficiency of light-dependent processes. Additional aspects of the technology relate to photo-selective effects on plant pests and diseases [117]. The ability of covers to absorb or reflect specific wavelengths can modify the red:far-red or red:blue ratios, which as noted before, can alter growth, architecture, and metabolism for better yield and fruit quality. An example of photo-selective covers that shift non-optimal wavelengths of sunlight to optimal wavelengths to maximize photosynthesis and growth is one of the Luminescent Light Emitting Agricultural Films (LLEAF) that shifts the green wavelength to the optimal red/far-red for plant utilization (https://www.lleaf.com/, accessed on 12 December 2022). These covers are currently being researched for their effects on the growth parameters of different crops.

The exploration of the impact of UV, LED, and light combinations and photo-selective covers on blueberry plant growth, yield and fruit quality could identify indoor farming practices to produce blueberries that provide maximal benefit to growers and consumers.

## 8. Mild Abiotic Stress Interventions to Enhance Antioxidant Capacity in Fruits

Plants are exposed to constantly changing environments and have therefore evolved homeostatic mechanisms that rely on sensing external signals for adaptation and survival. Abiotic stresses, such as high and low temperatures, water deficit, salinity, nutrient deficiency and high light intensity, are perceived by sensors localised either at the plasma membrane or within the cytoplasm, often resulting in the influx of Ca^2+^ or conversion of a protein’s activity state from one form to the other. These triggers in turn initiate signalling cascades, resulting in downstream cellular, physiological and morphological responses [118].

All abiotic stresses trigger the production of ROS (^1^O_2_, O_2_^•−^, H_2_O_2_, ^•^OH) as a consequence of disruptions in metabolic activity and leakage of electrons onto O_2_ from the electron transport activities of cell organelles [119]. For example, the osmotic stress and ion toxicity caused by salinity inhibits carbon fixation by reducing the availability of CO_2_, leading to excessive excitation energy and over-reduction of the electron transport system in the chloroplast and finally to increased generation of ROS. Plants respond by inducing non-enzymatic and enzymatic antioxidative systems to scavenge and break down harmful radicals, respectively. The first mechanism includes the accumulation of flavonoids, carotenoids, and vitamins such as ascorbic acid (vitamin C) and α-tocopherol (vitamin E), while the latter system includes the induction of enzymes such as peroxidases, superoxide dismutase, glutathione reductase, and others. The types of secondary metabolites, such as phenolic compounds, flavonoids and anthocyanins, produced by plants and their concentrations vary across species and to some extent across cultivars and cultivars within the same species [120]. The deposition of secondary metabolites also depends on the severity of stress and its duration, both of which can influence growth, yield and fruit quality [120,121].

The influence of salinity on secondary metabolite accumulation in different plant species has been reviewed [121], and the opportunity to enhance the nutrient quality of vegetables using salinity as a variable has been discussed previously [122]. Therefore, only a few examples are presented here. Peppers grown in moderately saline waters (15 mM) in a greenhouse were enhanced in antioxidants when harvested in the red state [123]. Increases of 30%, 58%, and 47% in total polyphenol content, total flavonoid content and total antioxidant capacity, respectively, were achieved as a response to 100 mM NaCl in leaves of *Amaranthus tricolor* [124]. Tomato plants grown in soilless nutrient solution with an electrical conductivity from 2.2 to 6.5 dS m^−1^ produced fruit with increased levels of β-carotene, lycopene, vitamin C and total phenolic content [125]. Other quality traits in tomatoes such as fruit dry matter, total soluble solids and lycopene contents were enhanced in response to the higher proportion of potassium (K) in the nutrient solution [125]. Strawberry plants grown in coir substrate and subjected to salt stress during fruit production had improved fruit nutritional value through higher contents of antioxidant compounds and soluble solids and reduced acidity [126].

Water deficit or drought stress enhanced secondary metabolite content (polyphenolic compounds, carotenoids, and vitamin C) in blueberry [73,127], sugar content in strawberry [128], and fruit aroma in strawberry and tomato [129,130]. In tomatoes, water deficit applied during the red stage of fruit ripening had the highest impact on fruit quality (soluble sugars, organic acids, aroma and vitamin C) as compared to mature green or orange stages [129]. Thus, the fruit developmental stage at which stress is applied is an important determinant of fruit quality response [127]. Blueberries are known to be sensitive to both salinity and water stress, although salinity at a certain level increased fruit size [131].

Temperature variations can strongly impact metabolic regulation in plants that are directed towards gaining thermostability of membranes, the cell wall, macromolecules, and their interactions. For example, plant metabolism in autumn is geared towards the synthesis of sugar alcohols, soluble sugars and low-molecular-weight nitrogenous compounds such as proline and glycine betaine, all of which function as cryoprotectants [132]. Positively charged organic molecules such as polyamines (spermine, spermidine, and putrescine) that function in cell proliferation and differentiation and that possess antioxidant properties are also induced in response to cold stress [133]. Moderate cold stress in blueberry induced an accumulation of antioxidant phenolic compounds, similar to those in tomato, watermelon [134], and suspension cultures of strawberry [135], whereas in spinach, cold stress induced higher vitamin C content [136]. Heat stress has a significant effect on ROS production and needs counteracting by antioxidant systems [137]. As a result, tomato plants exposed to 35 °C had significant increases in total phenolic content [134].

Although varieties have been bred to grow in milder climates, blueberries are native to areas with cold winters. The northern highbush varieties require winter chilling hours to break bud dormancy and produce fruit, whereas rabbiteye and southern highbush do not have a chilling requirement to produce fruit. There exists an opportunity to investigate whether or not the latter varieties have lower antioxidant potential than the chill-requiring varieties and if their antioxidant potential can be induced by exposure to mild cold stress.

In addition to air temperature, root zone temperature can alter plant growth and development by influencing the shoot apical meristem, hormonal balance, and water and mineral uptake [138,139]. In general, increasing root zone temperature from 12 to 25 °C promotes water and nutrient uptake functions of the root, and from 25 to 30 °C increases the root-to-shoot ratio. However, temperatures above 30 °C are detrimental to plant growth. Lowering root zone temperature promotes bioactive metabolite accumulation, such as higher concentrations of carotenoids in hydroponically grown carrots, vitamin C in spinach leaves and tomato fruit, and sugars in the leaves of red leaf lettuce and spinach [139,140,141,142]. Whilst root zone chilling is used as a technique by commercial growers to grow cool season crops in tropical climates, it also appears to be a promising approach for improving the nutritional value of vegetable and fruit crops grown hydroponically. Thus, water deficit and salinity (increase in nutrient solution electrical conductivity) stresses, which are first encountered by roots, along with root zone chilling, are root zone manipulating approaches that can be used to enhance nutritional value, boost flavour and aroma, and alter flowering and fruiting times of crops grown hydroponically.

While numerous pilot studies have shown that physiological interventions have the potential to increase the polyphenol content of fruits, with our current knowledge in blueberry, such an approach remains a challenge, as blueberry genotypes show significant location and genotype x environmental interactions [143]. Furthermore, protected cultivation of blueberry, compared to the open field, has shown reduced total flavonoid content [73], while the conventionally grown blueberries were seen to have 22% higher total polyphenol content than the organically grown blueberries [144]. Thus, in order to develop practical applications of mild abiotic stresses for increasing beneficial phytochemicals in blueberry under a cultivation system, it is crucial to identify (1) the ideal developmental stage when the crop plant can be exposed to stress, (2) the level of stress intensity, and (3) the duration of stress that can be applied without compromising other quality traits.

## 9. Blueberry Breeding and Genomics

The primary gene pool for blueberry consists of the three commonly cultivated species; *V. corymbosum, V. angustifolium,* and *V. virgatum* [145]. In the early 1900s, breeding programs focussed on expansion of highbush blueberry across temperate climates of North America, and by the 1940s, the objective was to extend the geographic range to warmer climates of the southern US. This was accomplished through crosses between northern highbush and wild southern species to yield southern highbush varieties [19,145]. In addition to their suitability for production in warmer climates, the southern highbush varieties are drought tolerant and have superior fruit qualities such as larger fruit size with improved texture and flavour. In this respect, *V. darrowii*, native to the southern US, has been a major contributor to traits such as evergreen, intense flavour, high bloom, and fruit stability under warm weather [18]. Since the 1970s, several southern highbush blueberry cultivars have been developed worldwide using the initially developed germplasm [146]. A large number of private breeding programs are mixing southern and northern germplasms to produce southern highbush types with chilling requirements spanning 0–750 h. For example, in Australia, Mountain Blue generated the important northern highbush ‘Brigitta’ hybrid, which due to high productivity and high-quality berries with excellent keeping quality, is grown all around the world. The breeding programs of the three large commercial growers in Australia, Driscoll, Costa and Mountain Blue are focussed on generating very low-chill and evergreen types together with desirable fruit traits [18]. The most sought-after traits by blueberry breeders are larger size, better flavour, increased bloom (coating of wax), easy detachment of fruit for harvesting by hand or machine, minimum scarring where fruit detaches from the pedicel, firmness, and long shelf life [14,19]. The second set of priorities for the blueberry industry include disease and pest resistance and abiotic stress tolerance traits [14]. Development of new high-quality cultivars while maintaining the desired traits that are useful for growers and wholesalers is both a challenge and an opportunity for blueberry breeders [145].

Traditional breeding of blueberry takes 10 to 20 years from performing the original cross to releasing a superior cultivar [19,147,148]. Modern breeding approaches utilize techniques, such as qualitative trait loci (QTLs), which are mapped using molecular markers that are correlated with an observed trait [149]. QTLs for chilling requirement and cold hardiness have been identified in a diploid blueberry population [150], and efforts are underway to identify QTLs for growth and fruit quality traits [151]. Some of these traits have also been evaluated in a tetraploid *V. corymbosum* mapping population, which could be used for QTL analyses in the future [143]. Candidate QTL regions for firmness, and putatively for machine harvest-related traits, have recently been reported in *V. corymbosum* [152]. Assuming diploid and tetraploid inheritance, a genome-wide association study (GWAS) analysis for fruit-related traits was conducted in southern highbush blueberry. Significantly associated single nucleotide polymorphisms (SNPs) with fruit quality traits were identified in both diploid [153] and tetraploid blueberry [152].

Marker-assisted selection (MAS) is a technique that uses DNA markers associated with specific traits to select plants for inclusion in breeding programs. The main advantage of MAS is the reduction in time to select the desired traits across the segregating progeny. Due to the long generation times and high levels of heterozygosity, MAS would be especially useful in breeding programmes for blueberries and other woody perennials. Recently, the first chromosome-scale genome assembly of tetraploid highbush blueberry (cultivar with high antioxidant levels), along with gene expression analysis to identify fruit metabolite-associated candidate genes, was reported [154]. A reference quality genome assembly is also available online for *V. darrowii* (https://phytozome-next.jgi.doe.gov/info/Vdarrowii_v1_2, accessed 20 October 2022). The availability of the reference genome should speed up the discovery and analysis of genes encoding fruit quality traits and the development of functional markers for MAS in blueberry breeding.

For understanding tissue, spatial and temporal expression patterns of genes, constructing genome maps and discovering new genes, the generation and analysis of short stretches of complementary DNA (typically < 1000 bp), termed as expressed sequence tags (ESTs), have proven advantageous [155,156]. The majority of the blueberry ESTs produced between 2003 and 2010 were derived from flower buds exposed to cold, with the aim of understanding cold acclimation in blueberry. Subsequently, the collection was expanded to include ESTs from roots and fruits at different developmental stages, those related to flavonoid metabolism, and those responsive to minerals in soil [157]. The first transcriptome sequences from leaves, flower buds at different stages of cold acclimation, and different developmental staged fruits were obtained for the highbush cultivar ‘Bluecrop’ [158]. Transcriptome data focussed on understanding the molecular mechanism involved in anthocyanin biosynthesis were obtained using cDNA libraries prepared from skin and pulp of blueberry fruit of the ‘Northland’ cultivar with high antioxidant capacity [159]. This dataset was expanded through transcriptome and gene expression profiling across berry development, especially during colour development, to gain better insight into key genes involved in anthocyanin biosynthesis [160]. More recently, transcriptomes of rabbiteye blueberry were generated from cDNA libraries prepared from fruits of plants with and without the epicuticular waxy coating on the fruit [161]. Although much remains to be accomplished in developing genetic and genomic resources in blueberry, the interest in linking genetic diversity in blueberry with antioxidant properties is growing, and a number of EST and genomic markers associated with the trait have been identified [162]. To facilitate progress in breeding of *Vaccinium* species, including blueberry, the Genome Database for *Vaccinium* (GDV, https://www.vaccinium.org, accessed 20 October 2022) has been set up to house genetic, breeding and genomic data (germplasms, genes and transcripts, genomes, genetic maps and markers, QTLs) [163,164]. Additional genomic resources are expected to soon become available through the Vaccinium Coordinated Agricultural Project (VacCAP), a US nation-wide transdisciplinary project (https://www.vacciniumcap.org, accessed on 20 October 2022).

While information linking fruit secondary metabolites with key genes controlling their accumulation is limited, the need to make this a priority in research has been recognised [145]. Following identification of key candidate genes, genetic engineering and gene editing approaches can be used in the enhancement of fruit quality traits. Success with stably transforming and regenerating blueberry plants has allowed transgenic blueberries to be developed with traits, such as yield increase and early flowering [165], herbicide resistance [166] and freezing tolerance [167]. Furthermore, the utility of using CRISPR/Cas9-mediated gene editing in blueberry for functional analysis of genes has been confirmed [168], suggesting that the application of this approach towards high-precision blueberry breeding is now only a matter of time.

## 10. Conclusions

**Figure 4:**
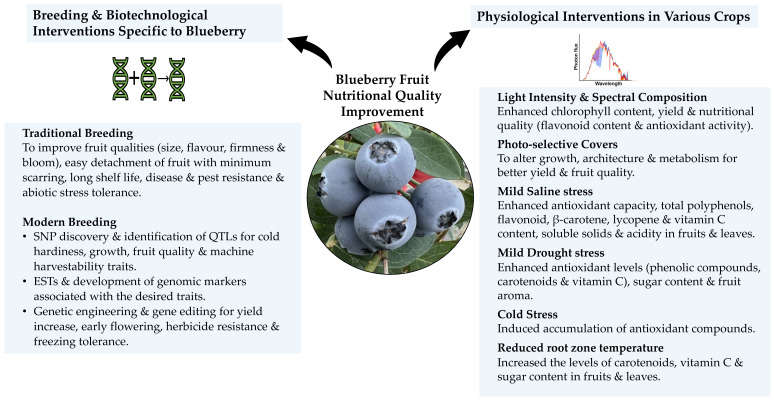
Summary of physiological and genetic interventions that could be used to optimise the nutritional quality of blueberry fruit grown under covers, along with current breeding efforts focused on other fruit traits.

Blueberry sales are forecast to significantly grow over the next few years due to increasing demand from developed and emerging markets. Major challenges in the production of blueberries are the short season for fresh blueberries and susceptibility to environmental conditions and mechanical damage. The adoption of polytunnels and hydroponic systems for blueberry cultivation has allowed these challenges to be mostly overcome, while giving better control over plant growth and irrigation management. However, blueberry cultivation under covers is associated with compromised light intensity and quality, which are important parameters in the synthesis of health-benefitting, antioxidant phytochemicals in blueberry fruit. A good understanding of the impact of growing conditions within polytunnels, compared to open-field, on blueberry fruit nutritional quality will allow for the development of methods based on light technology (UV-B, blue radiation, LLEAF covers) and mild stress treatments (water deficit, salinity, low temperature) to enhance antioxidants in blueberry fruit (summarised in Figure 4). With major efforts focused on developing genomic and genetic resources of blueberry, varietal development focused on nutritional quality of fruit will be possible in the future. Finally, an integrated approach of suitable cultivars with optimal lighting or discontinuous mild stress treatment can be used for enhancing nutraceutical traits in blueberry fruits grown under crop covers in sustainable hydroponic systems.
